# 3,3,4,4-Tetra­fluoro-2,3,4,5-tetra­hydro-1,6-benzodioxocine-8-carbaldehyde

**DOI:** 10.1107/S1600536810014133

**Published:** 2010-04-24

**Authors:** Zhuo Zeng, Jun-Wen Zhong, Hui Wang, Jin Wang, Wan-Wan Cao

**Affiliations:** a School of Chemistry and Environment, South China Normal University, Guangzhou 510006, People’s Republic of China

## Abstract

In the title compound, C_11_H_8_F_4_O_3_, the eight-membered dialk­oxy ring adopts a highly puckered conformation. In the crystal, mol­ecules are linked by weak C—H⋯O inter­actions.

## Related literature

For the applications of fluorinated mcarocyles, see: Babudri *et al.* (2007 ▶).
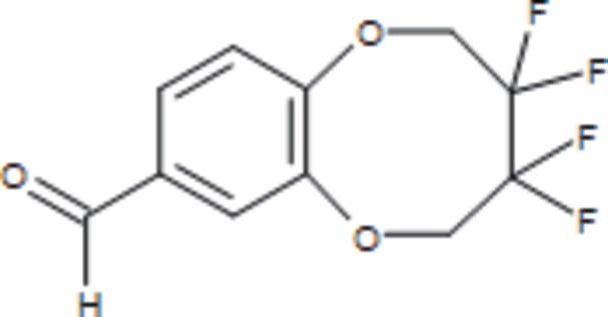


## Experimental

### 

#### Crystal data

C_11_H_8_F_4_O_3_
*M* *_r_* = 264.17
Monoclinic, 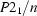

*a* = 9.142 (5) Å
*b* = 11.4935 (14) Å
*c* = 10.928 (10) Å
β = 104.109 (15)°
*V* = 1113.6 (12) Å^3^
*Z* = 4
Mo *K*α radiation
μ = 0.16 mm^−1^
*T* = 298 K
0.35 × 0.24 × 0.11 mm

#### Data collection

Bruker SMART CCD diffractometer
5552 measured reflections
2000 independent reflections
972 reflections with *I* > 2σ(*I*)
*R*_int_ = 0.044

#### Refinement

*R*[*F* ^2^ > 2σ(*F* ^2^)] = 0.047
*wR*(*F* ^2^) = 0.146
*S* = 0.97
2000 reflections
164 parameters
H-atom parameters constrained
Δρ_max_ = 0.15 e Å^−3^
Δρ_min_ = −0.15 e Å^−3^

### 

Data collection: *SMART* (Bruker, 2000 ▶); cell refinement: *SAINT* (Bruker, 2000 ▶); data reduction: *SAINT*; program(s) used to solve structure: *SHELXS97* (Sheldrick, 2008 ▶); program(s) used to refine structure: *SHELXL97* (Sheldrick, 2008 ▶); molecular graphics: *SHELXTL* (Sheldrick, 2008 ▶); software used to prepare material for publication: *SHELXTL*.

## Supplementary Material

Crystal structure: contains datablocks global, I. DOI: 10.1107/S1600536810014133/hb5362sup1.cif

Structure factors: contains datablocks I. DOI: 10.1107/S1600536810014133/hb5362Isup2.hkl

Additional supplementary materials:  crystallographic information; 3D view; checkCIF report

## Figures and Tables

**Table 1:** Hydrogen-bond geometry (Å, °)

*D*—H⋯*A*	*D*—H	H⋯*A*	*D*⋯*A*	*D*—H⋯*A*
C3—H3⋯O3^i^	0.93	2.52	3.193 (5)	130
C8—H8*B*⋯O3^ii^	0.97	2.43	3.343 (6)	157

Symmetry codes: (i) 
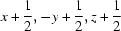
; (ii) 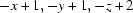.

**Fig. 1.**
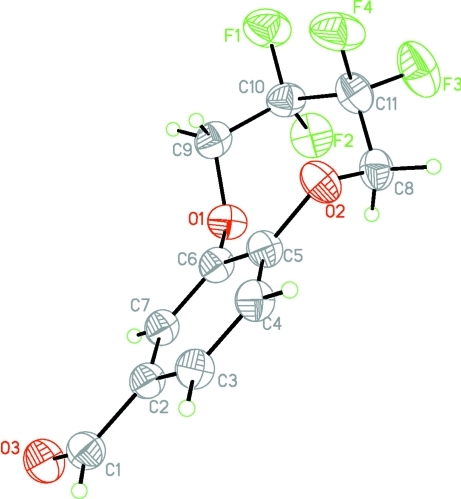
View of (I) showing displacement ellipsoids drawn at the 50% probability level.

## supplementary crystallographic information

### Comment

Fluorinated mcarocyles play an important role in the pharmaceutical, agrochemical and advanced materials fields (Babudri *et al.*, 2007). As part of our studies in this area, we now report the synthesis and structure of the title compound, (I).

The structure of this compound is shown in Fig. 1. The benzene ring is attached to a highly puckered eight-membered dialkoxy ring. The torsion angle at the fusion bond (O1—C8—C9—O2) is 3.27°. The C—C bond distances of the aromatic ring vary from 1.373 (3) to 1.407 (3) A, the latter being the C2—C7 fusion bond. The ring angles of the benzene ring also vary from 118.9 (8) to 120.8 (9)° indicating a slight distortion in this ring.

### Experimental

A mixture of 3,4-dihydroxy-benzaldehyde (0.345 g, 2.5 mmol), potassium carbonate (3.453 g, 25 mmol) was refluxed in acetonitrile (20 ml) at 373 K for 45 min, then a solution of methanesulfonic acid, trifluoro-, 2,2,3,3-tetrafluoro-1,4-butanediylester in acetonitrile (5 ml) was added, the mixture was heated under reflux for 12 h. After cooling to room temperature, the inorganic salts was removed by filtration . The filtrate was concentrated under reduced pressure, The residue was purified by flash chromatography on silica gel to afford the title compound as a white solid, yield 467 mg (70.8%).  Colourless blocks of (I) were grown by by slow evaporation from dichloromethane at room temperature.

### Refinement

H-atoms were placed in calculated positions with C—H = 0.93–0.97 Å and refined as riding atoms.

### Crystal data

**Table:**

C_11_H_8_F_4_O_3_	*F*(000) = 536.0
*M**_r_* = 264.17	*D*_x_ = 1.576 Mg m^−^^3^
Monoclinic, *P*2_1_/*n*	Mo *K*α radiation, λ = 0.71073 Å
Hall symbol: -P 2yn	Cell parameters from 733 reflections
*a* = 9.142 (5) Å	θ = 3.2–20.1°
*b* = 11.4935 (14) Å	µ = 0.16 mm^−^^1^
*c* = 10.928 (10) Å	*T* = 298 K
β = 104.109 (15)°	Block, colourless
*V* = 1113.6 (12) Å^3^	0.35 × 0.24 × 0.11 mm
*Z* = 4	

### Data collection

**Table:**

Bruker SMART CCD diffractometer	972 reflections with *I* > 2σ(*I*)
Radiation source: sealed tube	*R*_int_ = 0.044
graphite	θ_max_ = 25.2°, θ_min_ = 2.6°
ω scans	*h* = −6→10
5552 measured reflections	*k* = −13→13
2000 independent reflections	*l* = −13→11

### Refinement

**Table:**

Refinement on *F*^2^	Secondary atom site location: difference Fourier map
Least-squares matrix: full	Hydrogen site location: inferred from neighbouring sites
*R*[*F*^2^ > 2σ(*F*^2^)] = 0.047	H-atom parameters constrained
*wR*(*F*^2^) = 0.146	*w* = 1/[σ^2^(*F*_o_^2^) + (0.049*P*)^2^ + 0.4913*P*] where *P* = (*F*_o_^2^ + 2*F*_c_^2^)/3
*S* = 0.97	(Δ/σ)_max_ < 0.001
2000 reflections	Δρ_max_ = 0.14 e Å^−^^3^
164 parameters	Δρ_min_ = −0.15 e Å^−^^3^
0 restraints	Extinction correction: *SHELXL97* (Sheldrick, 2008), Fc^*^=kFc[1+0.001xFc^2^λ^3^/sin(2θ)]^-1/4^
Primary atom site location: structure-invariant direct methods	Extinction coefficient: 0.0047 (12)

### Special details

**Table:**

Geometry. All esds (except the esd in the dihedral angle between two l.s. planes) are estimated using the full covariance matrix. The cell esds are taken into account individually in the estimation of esds in distances, angles and torsion angles; correlations between esds in cell parameters are only used when they are defined by crystal symmetry. An approximate (isotropic) treatment of cell esds is used for estimating esds involving l.s. planes.
Refinement. Refinement of *F*^2^ against ALL reflections. The weighted *R*-factor wR and goodness of fit *S* are based on *F*^2^, conventional *R*-factors *R* are based on *F*, with *F* set to zero for negative *F*^2^. The threshold expression of *F*^2^ > σ(*F*^2^) is used only for calculating *R*-factors(gt) etc. and is not relevant to the choice of reflections for refinement. *R*-factors based on *F*^2^ are statistically about twice as large as those based on *F*, and *R*- factors based on ALL data will be even larger.

### Fractional atomic coordinates and isotropic or equivalent isotropic displacement parameters (Å^2^)

**Table:**

	*x*	*y*	*z*	*U*_iso_*/*U*_eq_	
F2	0.5814 (2)	0.90137 (19)	0.9443 (2)	0.0901 (8)	
F4	0.9709 (3)	0.8853 (2)	1.0613 (2)	0.1040 (9)	
F1	0.7668 (3)	0.9585 (2)	0.8674 (2)	0.1008 (9)	
F3	0.7960 (3)	0.9756 (2)	1.1293 (3)	0.1194 (10)	
C4	0.8950 (4)	0.4731 (3)	1.1272 (3)	0.0599 (10)	
H4	0.9789	0.4745	1.1953	0.072*	
C7	0.6454 (3)	0.4699 (3)	0.9227 (3)	0.0545 (9)	
H7	0.5614	0.4689	0.8547	0.065*	
C6	0.7048 (3)	0.5746 (3)	0.9715 (3)	0.0535 (9)	
C5	0.8301 (3)	0.5770 (3)	1.0754 (3)	0.0552 (9)	
C2	0.7096 (3)	0.3650 (3)	0.9738 (3)	0.0549 (9)	
C3	0.8354 (3)	0.3678 (3)	1.0779 (3)	0.0590 (10)	
H3	0.8785	0.2986	1.1136	0.071*	
C10	0.7221 (4)	0.8729 (4)	0.9373 (4)	0.0685 (11)	
C8	0.8155 (4)	0.7738 (3)	1.1564 (3)	0.0669 (11)	
H8A	0.8542	0.7957	1.2441	0.080*	
H8B	0.7108	0.7509	1.1445	0.080*	
C1	0.6488 (4)	0.2520 (4)	0.9223 (4)	0.0664 (11)	
H1	0.6927	0.1853	0.9636	0.080*	
C11	0.8265 (4)	0.8762 (4)	1.0718 (4)	0.0741 (11)	
C9	0.7179 (4)	0.7596 (3)	0.8678 (3)	0.0657 (10)	
H9A	0.6716	0.7710	0.7788	0.079*	
H9B	0.8197	0.7310	0.8761	0.079*	
O2	0.9011 (2)	0.6785 (2)	1.1261 (2)	0.0670 (7)	
O1	0.6327 (2)	0.6767 (2)	0.9195 (2)	0.0622 (7)	
O3	0.5453 (3)	0.2397 (2)	0.8295 (3)	0.0825 (9)	

### Atomic displacement parameters (Å^2^)

**Table:**

	*U*^11^	*U*^22^	*U*^33^	*U*^12^	*U*^13^	*U*^23^
F2	0.0728 (15)	0.0924 (18)	0.0987 (18)	0.0255 (13)	0.0085 (12)	0.0057 (14)
F4	0.0709 (15)	0.112 (2)	0.116 (2)	−0.0292 (14)	−0.0040 (13)	0.0202 (16)
F1	0.1117 (19)	0.0856 (18)	0.0951 (19)	−0.0162 (14)	0.0061 (14)	0.0252 (14)
F3	0.157 (2)	0.0757 (18)	0.103 (2)	0.0154 (16)	−0.0126 (17)	−0.0179 (16)
C4	0.049 (2)	0.073 (3)	0.046 (2)	−0.0005 (19)	−0.0088 (15)	0.0038 (19)
C7	0.0456 (19)	0.070 (3)	0.0410 (19)	−0.0046 (18)	−0.0020 (15)	0.0014 (18)
C6	0.0420 (19)	0.067 (3)	0.046 (2)	0.0014 (17)	0.0012 (16)	0.0051 (18)
C5	0.0449 (19)	0.065 (3)	0.049 (2)	−0.0042 (17)	−0.0011 (16)	−0.0010 (18)
C2	0.049 (2)	0.065 (2)	0.047 (2)	−0.0067 (17)	0.0062 (16)	−0.0003 (18)
C3	0.056 (2)	0.065 (3)	0.049 (2)	0.0014 (18)	0.0010 (17)	0.0049 (18)
C10	0.065 (2)	0.070 (3)	0.068 (3)	−0.003 (2)	0.010 (2)	0.013 (2)
C8	0.063 (2)	0.079 (3)	0.052 (2)	−0.001 (2)	0.0015 (17)	−0.009 (2)
C1	0.064 (2)	0.071 (3)	0.063 (2)	−0.010 (2)	0.012 (2)	−0.003 (2)
C11	0.070 (3)	0.071 (3)	0.074 (3)	0.000 (2)	0.004 (2)	−0.009 (2)
C9	0.058 (2)	0.084 (3)	0.049 (2)	0.000 (2)	0.0007 (16)	0.011 (2)
O2	0.0481 (13)	0.0678 (17)	0.0733 (18)	−0.0011 (12)	−0.0077 (12)	−0.0091 (13)
O1	0.0472 (13)	0.0663 (17)	0.0647 (16)	0.0009 (12)	−0.0025 (11)	0.0115 (13)
O3	0.0749 (17)	0.096 (2)	0.0663 (18)	−0.0205 (16)	−0.0032 (14)	−0.0142 (15)

### Geometric parameters (Å, °)

**Table:**

F2—C10	1.347 (4)	C2—C1	1.469 (5)
F4—C11	1.357 (4)	C3—H3	0.9300
F1—C10	1.368 (4)	C10—C9	1.504 (5)
F3—C11	1.365 (4)	C10—C11	1.545 (6)
C4—C3	1.381 (4)	C8—O2	1.432 (4)
C4—C5	1.392 (4)	C8—C11	1.515 (5)
C4—H4	0.9300	C8—H8A	0.9700
C7—C6	1.374 (4)	C8—H8B	0.9700
C7—C2	1.397 (4)	C1—O3	1.215 (4)
C7—H7	0.9300	C1—H1	0.9300
C6—O1	1.398 (4)	C9—O1	1.430 (4)
C6—C5	1.402 (4)	C9—H9A	0.9700
C5—O2	1.383 (4)	C9—H9B	0.9700
C2—C3	1.407 (4)		
			
C3—C4—C5	120.3 (3)	O2—C8—C11	109.4 (3)
C3—C4—H4	119.8	O2—C8—H8A	109.8
C5—C4—H4	119.8	C11—C8—H8A	109.8
C6—C7—C2	120.9 (3)	O2—C8—H8B	109.8
C6—C7—H7	119.6	C11—C8—H8B	109.8
C2—C7—H7	119.6	H8A—C8—H8B	108.3
C7—C6—O1	118.4 (3)	O3—C1—C2	124.6 (4)
C7—C6—C5	119.9 (3)	O3—C1—H1	117.7
O1—C6—C5	121.6 (3)	C2—C1—H1	117.7
O2—C5—C4	116.7 (3)	F4—C11—F3	106.6 (3)
O2—C5—C6	123.5 (3)	F4—C11—C8	108.8 (3)
C4—C5—C6	119.7 (3)	F3—C11—C8	108.5 (4)
C7—C2—C3	119.0 (3)	F4—C11—C10	108.1 (4)
C7—C2—C1	121.8 (3)	F3—C11—C10	108.1 (3)
C3—C2—C1	119.2 (3)	C8—C11—C10	116.4 (3)
C4—C3—C2	120.1 (3)	O1—C9—C10	109.0 (3)
C4—C3—H3	119.9	O1—C9—H9A	109.9
C2—C3—H3	119.9	C10—C9—H9A	109.9
F2—C10—F1	106.1 (3)	O1—C9—H9B	109.9
F2—C10—C9	109.4 (3)	C10—C9—H9B	109.9
F1—C10—C9	108.4 (3)	H9A—C9—H9B	108.3
F2—C10—C11	108.5 (4)	C5—O2—C8	120.5 (2)
F1—C10—C11	108.3 (3)	C6—O1—C9	118.1 (2)
C9—C10—C11	115.8 (3)		
			
C2—C7—C6—O1	−177.7 (3)	F2—C10—C11—F4	−160.5 (3)
C2—C7—C6—C5	−1.1 (5)	F1—C10—C11—F4	−45.8 (4)
C3—C4—C5—O2	−176.8 (3)	C9—C10—C11—F4	76.1 (4)
C3—C4—C5—C6	−0.9 (5)	F2—C10—C11—F3	−45.4 (5)
C7—C6—C5—O2	176.6 (3)	F1—C10—C11—F3	69.2 (4)
O1—C6—C5—O2	−6.8 (5)	C9—C10—C11—F3	−168.8 (3)
C7—C6—C5—C4	1.0 (5)	F2—C10—C11—C8	76.9 (4)
O1—C6—C5—C4	177.6 (3)	F1—C10—C11—C8	−168.5 (3)
C6—C7—C2—C3	0.9 (5)	C9—C10—C11—C8	−46.5 (5)
C6—C7—C2—C1	−179.4 (3)	F2—C10—C9—O1	−48.7 (4)
C5—C4—C3—C2	0.8 (5)	F1—C10—C9—O1	−163.9 (2)
C7—C2—C3—C4	−0.8 (5)	C11—C10—C9—O1	74.2 (4)
C1—C2—C3—C4	179.5 (3)	C4—C5—O2—C8	−134.3 (3)
C7—C2—C1—O3	3.8 (6)	C6—C5—O2—C8	49.9 (5)
C3—C2—C1—O3	−176.6 (4)	C11—C8—O2—C5	−114.5 (3)
O2—C8—C11—F4	−41.8 (4)	C7—C6—O1—C9	−121.7 (3)
O2—C8—C11—F3	−157.5 (3)	C5—C6—O1—C9	61.7 (4)
O2—C8—C11—C10	80.4 (4)	C10—C9—O1—C6	−120.0 (3)

### Hydrogen-bond geometry (Å, °)

**Table:**

*D*—H···*A*	*D*—H	H···*A*	*D*···*A*	*D*—H···*A*
C3—H3···O3^i^	0.93	2.52	3.193 (5)	130
C8—H8B···O3^ii^	0.97	2.43	3.343 (6)	157

Symmetry codes: (i) *x*+1/2, −*y*+1/2, *z*+1/2; (ii) −*x*+1, −*y*+1, −*z*+2.
